# A Novel Mutation in the *RPE65* Gene Causing Leber Congenital Amaurosis and Its Transcriptional Expression *In Vitro*

**DOI:** 10.1371/journal.pone.0112400

**Published:** 2014-11-10

**Authors:** Guoyan Mo, Qin Ding, Zhongshan Chen, Yunbo Li, Ming Yan, Lijing Bu, Yanping Song, Guohua Yin

**Affiliations:** 1 China Key Laboratory of TCM Resource and Prescription, Hubei University of Chinese Medicine, Ministry of Education, Wuhan 430065, China; 2 Department of Ophthalmology, Wuhan General Hospital of Guangzhou Military Command, Wuhan 430070, China; 3 Beijing University of Chinese Medicine Third Affiliated Hospital, Beijing 100029, China; 4 Department of Biology, University of New Mexico, Albuquerque, NM, 87131, United States of America; 5 Department of Plant Biology and Pathology, Rutgers, The State University of New Jersey, New Brunswick, NJ, 08901, United States of America; 6 Wuhan Sheng Da An Biotech Service Co. Ltd., Wuhan, China; Justus-Liebig-University Giessen, Germany

## Abstract

The retinal pigment epithelium-specific 65 kDa protein is an isomerase encoded by the *RPE65* gene (MIM 180069) that is responsible for an essential enzymatic step required for the function of the visual cycle. Mutations in the *RPE65* gene cause not only subtype II of Leber congenital amaurosis (LCA) but also early-onset severe retinal dystrophy (EOSRD). This study aims to investigate a Chinese case diagnosed as EOSRD and to characterize the polymorphisms of the *RPE65* gene. A seven-year-old girl with clinical symptoms of EOSRD and her parents were recruited into this study. Ophthalmologic examinations, including best-corrected visual acuity, slit-lamp, Optical coherence tomography (OCT), and fundus examination with dilated pupils, were performed to determine the clinical characteristics of the whole family. We amplified and sequenced the entire coding region and adjacent intronic sequences of the coding regions of the *RPE65* gene for the whole family to explore the possible mutation. Our results demonstrate that the patient exhibited the typical clinically features of EOSRD. Her bilateral decimal visual acuity was 0.3 and 0.4 in the left and right eyes, respectively. Spectral-domain optical coherence tomography (SD-OCT) was used to assess the retinal stratification for the whole family. All together, we identified four mutations within the *RPE65* gene (c.1056G>A, c.1243+2T>A, c.1338+20A>C and c.1590C>A) in the patient. Among the four mutations, c.1056G>A and c.1338+20A>C had been reported previously and another two were found for the first time in this study. Her mother also carried the novel mutation (c.1243+2T>A). Either a single or a compound heterozygous or a homozygous one mutation is expected to cause EOSRD because mutations of *RPE65* gene usually cause an autosomal recessive disease. Therefore, we speculate that the c.1590C>A mutation together with the c.1243+2T>A mutation may cause the patient’s phenotype.

## Introduction

**Figure 1:**
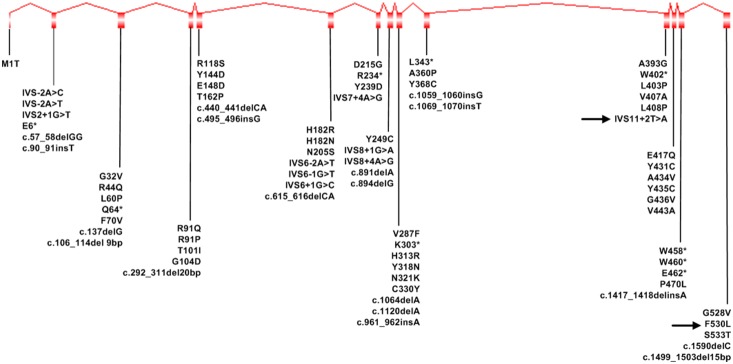
LCA related mutations in *RPE65* gene. The black arrow indicates the novel mutation discovered in this study.

Retinal pigment epithelium-specific 65 kDa protein (RPE65, GenBank accession No. NP000320.1) is an isomerase preferentially expressed in the retinal pigment epithelium (RPE) [1], [2]. It is responsible for retinol isomerization and converts all-*trans* retinyl ester to11-*cis* retinol in the visual cycle [3]–[5]. Previous research demonstrated that retinol isomerization was an essential enzymatic step required for functional vision [6], [7]. RPE65 is a microsomal protein encoded by *RPE65* gene (MIM 180069), containing 14 coding exons and localizing in chromosome 1p31 [8]. Mutations in *RPE65* gene were primarily reported in patients with Leber congenital amaurosis (LCA, MIM204000). Now, 86 mutations have been identified in the *RPE65* gene in patients with LCA [9]–[33]; we summarize these mutations in Figure 1.

LCA, first described in 1869 by Leber T [34], is a severe congenital or early infant-onset form of inherited retinal dystrophy [35]. In general, LCA is defined as blindness within the first two years of life. Based on previous descriptions of patients with LCA, this disease has a wide spectrum of presentation such as early severe visual deficits in childhood, the oculo-digital sign (habitually rubbing or poking the eyes), refractive errors, heterogeneity in retinal appearance, macular atrophy, and optic nerve pallor [36]. In addition, congenital onset and amaurosis by the second year of life are features that define LCA. Early-onset severe retinal dystrophy (EOSRD) is one of phenotype of all *RPE65* mutations. Unlike LCA, EOSRD is characterized by an amaurosis in the second decade and later and leaves a vision of 0.3 at the two-year age. The EOSRD has several names: juvenile and early-onset retinitis pigmentosa, childhood-onset severe retinal dystrophy, and severe early childhood onset retinal dystrophy (SECORD) [37]–[40]. Many studies indicated that LCA was extremely genetically heterogeneous and was associated with more than 17 genes. Moreover, many mutations associated with the inheritance of LCA have been described and used to differentiate the subtypes of LCA1∼15, for instance, *GUCY2D* (LCA1), *RPE65* (LCA2), *AIPL1* (LCA4), *RPGRIP1* (LCA6), *CRX* (LCA7), and *CRB1* (LCA8) (http://wwww.ncbi.nlm.nih.gov/omim). Among these known diseased-causing genes, *RPE65* mutations were first identified and their prevalence ranges from 1.7% to 16% in LCA cohorts in the United States, Canada, Saudi Arabia, Asia, and India [10], [41], [42]. To date, two cases of *RPE65* mutations with LCA have been reported in China [31], [32], while most cases occur in Western populations. Notably, homozygous and compound heterozygous mutations in *RPE65* gene are associated with subtype II of LCA or EOSRD [37], [43]. The description of EOSRD was coined for *RPE65* mutations.

In this study, we report the clinical examinations and genetic analysis of *RPE65* gene in a Chinese family. Sanger sequencing was used to analyze all the coding regions of the *RPE65* gene along with the adjacent intronic regions. Furthermore, we constructed the *RPE65 *minigene containing the c.1243+2T>A mutation and investigated the effects of that mutation in *in vitro* splicing.

## Materials and Methods

### Clinical data and sample collection

This study was reviewed by the Department of Ophthalmology, Wuhan General Hospital of Guangzhou Military Command on Clinical Investigation and it conformed to the tenets of the Declaration of Helsinki. The seven-year-old girl and her family were referred to the Department of Ophthalmology, Wuhan General Hospital of Guangzhou Military Command on Clinical Investigation. After informed consent was obtained, all participants underwent six ophthalmologic examinations, including best-corrected visual acuity, slit-lamp, and fundus examination with dilated pupils to exclude infection or other diseases. Ophthamoscopic findings were recorded by color fundus photography. Optical coherence tomography (OCT, Topcon 3D-1000 Mark II, Tokyo Japan) was used to examine the retinal structure. Spectral-domain OCT (SD-OCT) recording with 3D macular protocol was performed with 6-mm single line scans over the fovea. In detail, the 3D macular protocol consists of a radial-scanning composed of 512×128 scan resolution covering an area of 6×6 mm in the macular region. The patient underwent fundus fluorescein angiography (FFA) and visual field examination. Clinical diagnosis was based on the results of the above-mentioned ophthalmologic examinations.

### Mutation identification

Blood samples were obtained by venipuncture and genomic DNA was extracted according to the manufacturer’s protocol (TIANamp Blood DNA Kit, Tiangen) as described in our previous report [44]. For sequencing, the entire coding region and adjacent intronic sequences of 14 coding regions of the *RPE65* gene were amplified by PCR, using the primers in Table 1. PCR products were purified with the AxyPrep DNA Gel Extraction Kit (Axygen, CA, USA). All PCR products were bi-directionally sequenced with the dideoxy nucleotide chain terminator technique. Sequencing was performed on an automated sequencer – ABI 3730XL DNA Analyzer (ABI, USA). The results were assembled and analyzed using the Applied Biosystems Sequencing Analysis 5.2 software. Sequences were aligned with the published cDNA sequences of *RPE65* gene (GenBank accession no. NM_000329). We also assessed the potential functional consequences of nucleotide changes using multiple web servers for mutation analysis such as PolyPhen-2 (Polymorphism Phenotyping, http://genetics.bwh.harvard.edu/pph-2/) [45], SIFT (Sorting Intolerant From Tolerant, http://sift.jcvi.org/) [46], and Automated Splice Site and Exon Definition Analyses (http://splice.uwo.ca/) with default parameters.

**Table 1:** Primers used in the study.

Exons	Primers	Sequencing (5′→3′)
2	RPE65–2F	GCAGGAGTGAACAGGCTTTG
	RPE65–2R	AGAGACGCCAAGGAATAGGAA
3	RPE65–3F	GAGGGCTGGAAATGAAAATC
	RPE65–3R	ACATTGTGAGAAGAAAGTGGGTA
4/5	RPE65–45F	GGTCACCCCAAGAAAGTGAG
	RPE65–45R	GGATTTGAAACTTAATGTGGCTC
6	RPE65–6F	AACTCAAGGTGAAAGAGGGTAGA
	RPE65–6R	AGAGAACTTGGACACTTGCTTTC
7–9	RPE65–789F	GGAGAAAATGAAAATAACCCCTC
	RPE65–789R	GAGTGCAGCAGCTCTGTAAAA
10	RPE65–10F	GAATAAAGAACAGGCAGGCACT
	RPE65–10R	TTGCTTTTGCTAAGTCACAGTAC
11–13	RPE65–1123F	CCTCCCTGCATGTTGACCT
	RPE65–1123R	GCTCCATCGTGACACCAAAT
14	RPE65–14F	ATGCCAGGTGGTACAAGAGTCA
	RPE65–14R	TGCTCAACTCAGTGCTTTCTGTA
	F1*	GTC**GAATTC**GTCACGCTCCCCAATACAAC
	R1*	GCA**GTCGAC**GATGGGTTCTGATGGGTATG
	F2*	CTCGTCAAGGAGAGATGATCTAGAGAAAACTTCACACGGGAG
	R2*	TCTCTAGATCATCTCTCCTTGACGAGGCCCTG

Notes: *denotes primers for amplification of the wild and mutant fragments of exons 11, 12, and 13 of *RPE65* gene. Bold and underlined sequences are restriction enzyme sites.

### Cell line and cell culture

The 293T cell line was purchased from the Type Culture Collection of the Chinese Academy of Sciences (Wuhan, China) and cultured in Dulbecco’s modified Eagle’s medium (DMEM, GIBCO) supplemented with 2 mM glutamine, 10% fetal calf serum (FBS, GIBCO), 100 UI/ml penicillin, and 100 µg/ml streptomycin sulfate. The Cells were maintained at 37°C in a 5% CO_2_ incubator until confluent, then sub-cultured at 1∶3 to 1∶10 dilutions using trypsin-EDTA.

### RPE65 minigene construction

*RPE65* mutated minigenes (pCIneo-m65) were constructed by subcloning the c.1243+2T>A mutation from the patient into pCI-neo vector (Promega, Madison, WI). The wild-type minigene (pCIneo-65), without the c.1243+2T>A mutation, was generated by subcloning the genomic DNA of her father. PCR-amplified exons (11, 12, and 13) of the *RPE65* gene were inserted at the *Eco*RI/*Sal*I restriction enzyme site in pCI-neo vector. Primers and conditions used in the PCR amplification of the inserts were listed in Table 1. The corresponding DNA inserts were confirmed by sequencing.

### Transfection, RNA isolation, and RT-PCR

To analyze the effects of the c.1243+2T>A mutation on splicing *in vitro*, 293T cells were transfected with *RPE65 *minigenes. Prior to transfection, cells were seeded with a density of 4.0×10^5^ cells per well in a six-well plate and grown to approximately 80% confluence. *RPE65 *minigene used in this study was transfected into cells with Lipofectamine 2000 reagent (Invitrogen) by using 2 µg of DNA per well. After 24 h transfection, cells were serum starved for another 24 h before harvest. Total RNA was extracted from cells using TRIzol reagent (Invitrogen) according to the manufacturer’s instructions. DNase I (Promega) was used to treat the RNA extract to eliminate DNA contamination. 1.0 µg of treated RNA was used as a template for reverse transcription using the First Strand cDNA Synthesis Kit (TOYOBO). The exogenous *RPE65 *minigene transcript was amplified using F1 and R1 primers (Table 1) and the fragment sizes of the wild and mutant PCR products were detected by 2% agarose gels electrophoresis and stained with ethidium bromide. To confirm the nucleotide sequences, the wild and mutant PCR products were purified from the gel and sent for sequencing.

## Results

### Clinical features

**Figure 2:**
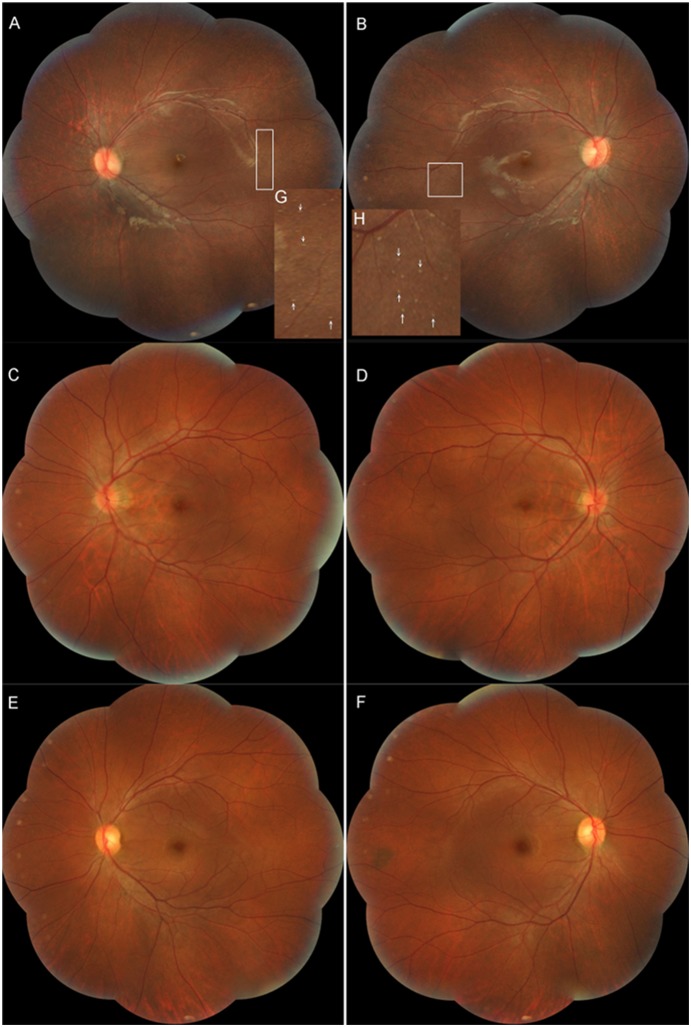
Fundus photographs of both eyes in the family. Color fundus photographs of both eyes (a: left eye; b: right eye) show mildly attenuated retinal vessels, some whitish dots, and numerous grayish deposits in the mid-peripheral retina of the patient. The inserted panels (g, h) show a magnification of the indicated areas. Whitish dots are marked by white arrowheads. Fundus photographs of her father (c, d) and her mother (e, f) show no whitish dots or grayish deposits in the mid-peripheral retina.

In this Chinese family, only the seven-year-old girl reported the typical characteristics of EOSRD. She was a primary school student. When she was three years old in 2008, her parents found she could not see toys in the dark, which meant her visual acuity was markedly decreased in dimmer conditions. Moreover, when she went inside from outdoor sunlight, her dark adaption took nearly two hours. In 2011, her eyesight problem made it difficult for her study at school. She was diagnosed as EOSRD by a full ophthalmologic examination in 2012. Her fundus examination demonstrated mildly attenuated retinal vessels, some whitish dots, and numerous grayish deposits in the mid-peripheral retina (Figure 2). However, no whitish dots or grayish deposits were observed on the fundi of her parents.

**Figure 3:**
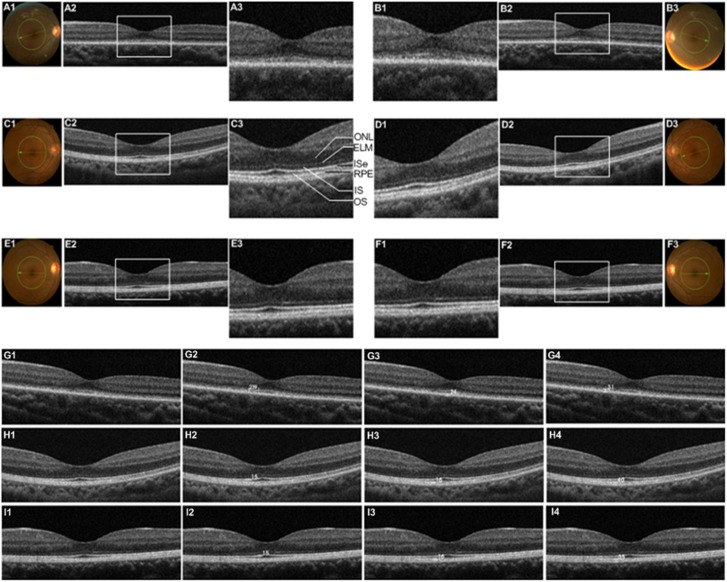
Foveal spectral-domain optical coherence tomography (SD-OCT) recordings of eyes in the whole family. The green circle in fundus photographies (a-1, b-3, c-1, d-3, e-1, and f-3) shows the scanning position of the presented SD-OCT recordings of the family using 3D macular protocol. Foveal SD-OCT pictures of the patients (a-2 and b-2) show abnormalities of retinal stratification including extremely thinned ONL (outer nuclear layer), heavily thinned RPE (retinal pigment epithelial) layer, altered photoreceptor layers (ELM, IS, ISe, and OS). The patient’s grayscale SD-OCT recordings (a-2, and b-2) show that the boundaries of ELM, IS, ISe and OS layers are not well demarcated and the ISe layer could hardly be discerned. The white box in grayscale SD-recordings (a-2, b-2, c-2, d-2, e-2, and f-2) indicates the area of enlargement. The enlargement of grayscale SD-OCT recordings at the fovea of both eyes (a-3, and b-1) show that the ELM, IS, ISe and OS layers are not continuous. The abnormalities of retinal stratification are not present on the foveal SD-OCT recordings of her father (c and d) or mother (e and f). We measured the thickness of ISe, RPE, and RPE-choroid of the patient and her parents’s right eyes (Figure 3g, 3h, and 3i). Her father/mother’ results were 15 µm/15 µm (Figure 3h-2 and 3i-2), 16 µm/16 µm (Figure 3h-3 and 3i-3), and 40 µm/35 µm (Figure 3h-4 and 3i-4). Because the boundaries of IS, ISe, OS, and RPE are not clear, we measured the noncontiguous area of ISe layer, RPE-choroid and ISe-choroid,and the results were 239 µm (Figure 3g-2), 24 µm (Figure 3g-3), and ISe-choroid (Figure 3g-4), respectively.

**Figure 4:**
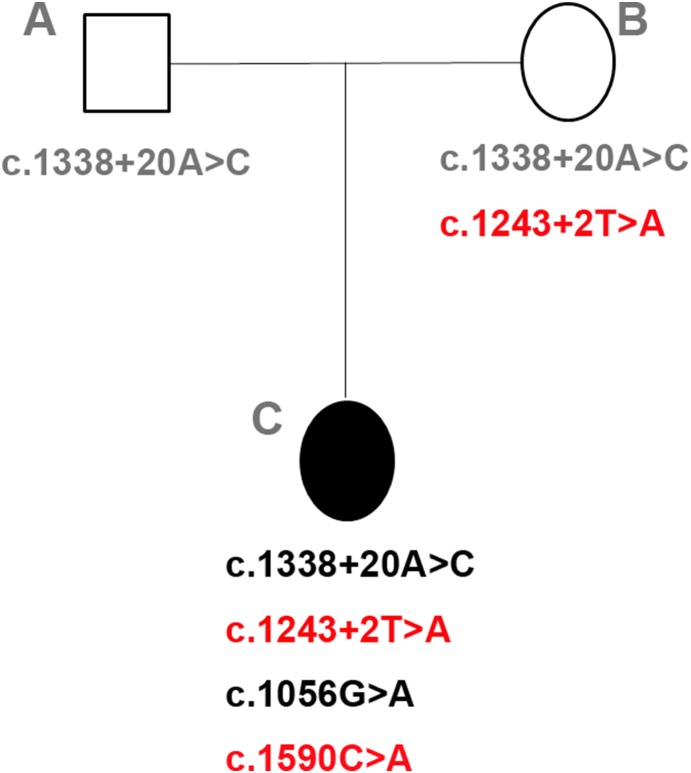
Pedigree of the Chinese family and mutations of *RPE65* gene. In the family structure, male and female are represented by squares and circles, respectively. The filled square symbol represents the ESORD-affected daughter (c). One mutation (c.1338+20A>C) is detected in *RPE65* gene of her father (a); two other mutations (c.1243+2T>A and c.1590C>A) are detected in her mother (b); four mutation are found in the daughter (a). The red color highlights the novel mutation.

The SD-OCT scanning with 3D macular protocol was used to examine the retinal stratification. Compared with foveal SD-OCT scanning recordings of her parents’ eyes, the patient’s foveal SD-OCT images of both eyes showed several characteristics of alterd retinal stratification including extremely thinned outer nuclear layer (ONL), heavily thinned retinal pigment epithelial (RPE) layer, altered photoreceptor layers including external limiting membrane (ELM), inner segment (IS), outer segments (OS), and inner segment elipsoid (ISe) (Figure 3a and 3b). In her patients’ grayscale SD-OCT recording (Figure 3 c-2, 3d-2, 3e-2, and 3f-2), the boundaries of the photoreceptor layers (ELM, IS, ISe, and OS) were not well demarcated. The enlargement of grayscale SD-OCT recordings at the fovea of her patients’ eyes (Figure 3 c-3, 3d-1, 3e-3, and 3f-1) showed that the ELM, IS, ISe and OS layers were not continuous and could not be clearly identified, and the ISe layer previously named IS/OS junction could hardly be shown. In addition, the boundaries of OS/RPE and RPE-Bruch’s membrane complex could not be discerned. But her parents’ foveal SD-OCT recordings showed that the boundaries of the retinal layers were well demarcated and the retinal layers could be clearly identified (Figure 3c-2, 3d-2, 3e-2, and 3f-2). The aforementioned abnormalities were not presented in her parents’ grayscale foveal SD-OCT recordings (Figure 3 c-2, 3d-2, 3e-2, and 3f-2). Meanwhile, we also measured the thinkness of ISe, RPE, and RPE-choroid of the patient and her parents’s right eyes (Figure 3g, 3h, and 3i). Her father/mother’ results were 15 µm/15 µm (Figure 3h-2 and 3i-2), 16 µm/16 µm (Figure 3h-3 and 3i-3), and 40 µm/35 µm (Figure 3h-4 and 3i-4). Because the boundaries of IS, ISe, OS, and RPE are not clear, we cannot measure the thickness of ISe and RPE. Therefore, we measured the noncontiguous area of ISe layer, RPE-choroid and ISe-choroid,and the results were 239 µm (Figure 3g-2), 24 µm (Figure 3g-3), and ISe-choroid (Figure 3g-4), respectively.

### *RPE65* gene mutation analysis

Based on the complete sequence analysis of the coding and adjacent intronic regions of *RPE65* gene, four mutations were detected in this family. These mutations included one missense, one silent, and two intronic changes. All mutations are listed in Table 2. All four mutations were present in the heterozygous state. The father carried only one point mutation (c.1338+20A>C, Figure 4), and the mother carried two point mutations (c.1243+2T>A and c.1338+20A>C, Figure 4). The daughter carried four mutant points: c.1338+20A>C (from her parents), c.1243+2T>A (from her mother), c.1056G>A, and c.1590C>A (Figure 4). Meanwhile, we also used mutiple web servers including PolyPhen, SIFT, and Automated Splice Site Analyses to perform the mutation analysis (Table 2). The loss of the splice site was predicted for the c.1243+2T>A mutation by Automated Splice Site Analyses; the c.1056G>A (rs12145904) mutation is predicted to be “benign” and “neutral” according to the results of PolyPhen and SIFT; the c.1590C>A (F530L) mutation is predicted to be “possibly damaging” and “deleterous” by PolyPhen and SIFT; the c.1338+20A>C (rs12564647) mutation cannot be evaluated by Polyphen and SIFT. The c.1338+20A>C (rs12564647) mutation is listed in the SNP database of GenBank (http://www.ncbi.nlm.nih.gov/projects/SNP/snp_ref.cgi?rs=12564647).

**Table 2:** The *RPE65* gene mutations in the seven-year-old patient.

Variants Description				Computational Prediction		References or Annotations	Daughter or Parent
Nucleotide Change	Amino Acid Change	State	Conservation	PolyPhen/Splice Site	SIFT		
c.1056G>A	E352E	Hetero	No	benign	neutral	rs12145904, [33]	daughter
c.1243+2T>A	Splicing change	Hetero	N/A	Splicing site abolished	N/A	this study	daughter, mother
c.1338+20A>C	N/A	Hetero	N/A	N/A	N/A	rs12564647, [33]	daughter, mother, father
c.1590C>A	F530L	Hetero	Yes	possibly damaging	deleterious	this study	daughter

Abbreviation: Hetero - Heterozygous, N/A - Not applicable.

### Transcriptional expression of *RPE65 *minigenes in 293T cells

**Figure 5:**
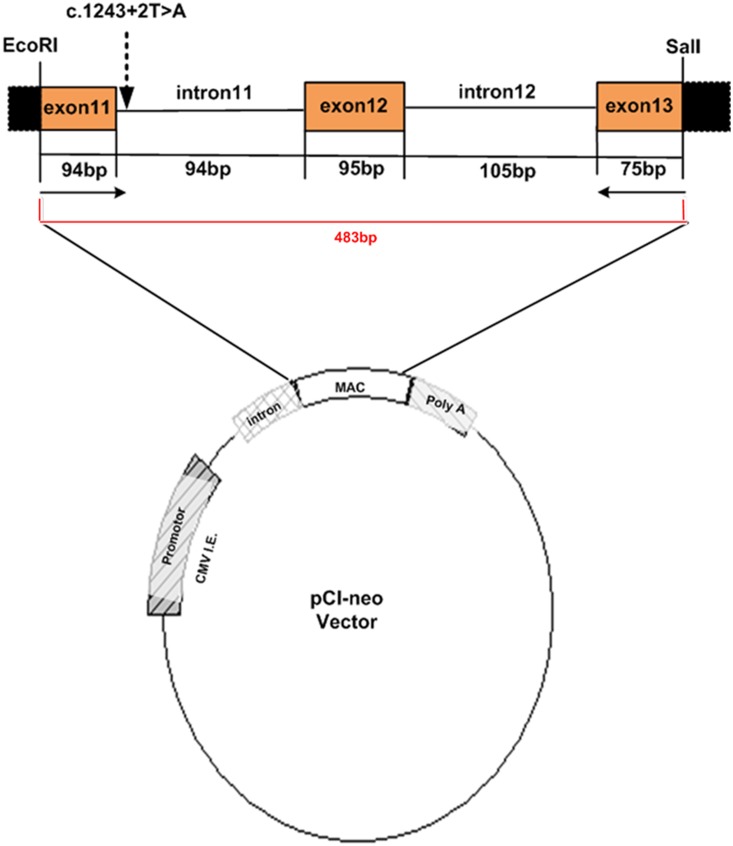
Structure of *RPE65 *minigene. The pCIneo minigenes of *RPE65* gene were constructed to contain three exons (exon 11, 12, and 13) and flanking intronic sequences (intron 11 and 12) from wild or mutant type (c.1243+2T>A) of *RPE65* gene. *Eco*RI and *Sal*I represent restriction enzyme sites. Horizontal arrows indicate the positions and the directions of the primers. The red 483 bp indicates the amplified product.

**Figure 6:**
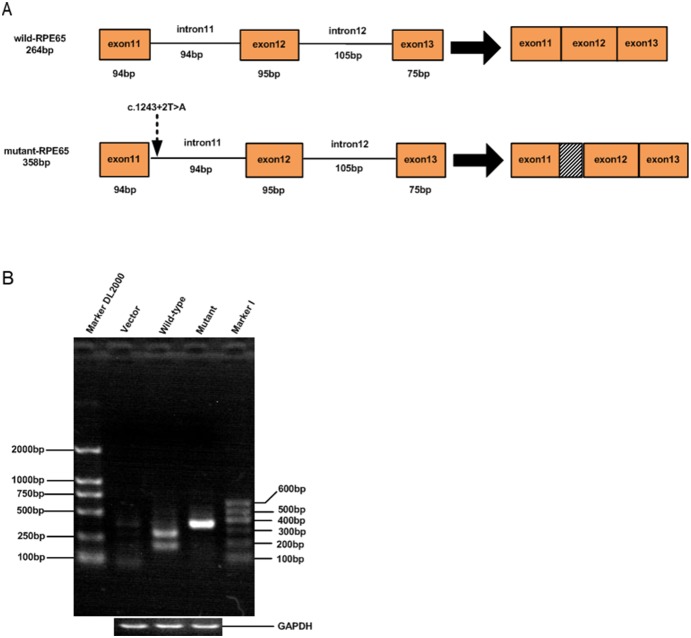
Analysis of pre-mRNA splicing of pCIneo minigenes in the transfected 293T cell line. A. Graphic representation of pre-mRNA splicing of wild type and mutant (C.1243+2T>A) minigenes of *RPE65* gene. B. The isolated RNA of transfected cells was amplified by RT-PCR analysis. The different splicing products for wild type (264 bp) and mutant (358 bp) are shown on a 2% agarose gel. The mutant lane demonstrated that only a 358 bp band was obtained from the mutant-RPE65 minigene (a). The wild-type lane showed two different size DNA bands: one is a 264 bp band, and another is ∼200 bp band. The 264 bp DNA band is the expected size (a). The 200 bp band is the amplification caused by mispriming.

To identify the effects of the c.1243+2T>A mutation in intron 11 of *RPE65* gene on splicing, we constructed two minigenes: the c.1243+2T>A mutation in *RPE65* gene (pCIneo-m65) and the wild type (pCIneo-65), and transfected them into 293T cell line. Total RNA was isolated from 293T cells transfected with the minigene constructs (Figure 5), and then used for RT-PCR to amplify the exons of 11, 12, and 13 of *RPE65* gene. PCR products indicating variations in splicing, were resolved and analyzed by a 2% agarose gel electrophoresis (Figure 6b). The RT-PCR product of the wild type showed a 264 bp DNA band, as the expected normal transcript (Figure 6a), but the mutant type produced a 358 bp band (Figure 6a). It is 94 bp longer than the normal transcript of 264 bp (Figure 6b), indicating an insertion of the complete intron 11 (Figure 6a). Sequence analysis of the product revealed that 94 bp were indeed inserted at the 3′ end of exon 11. The sequence of the 94 bp insertion perfectly matched that of intron 11 in *RPE65* gene containing the c.1243+2T>A mutation. Interestingly, there was another smaller size DNA band (about 200 bp) in wild-type (Figure 6b) not present in mutant. This could be caused by mispriming. These results show that the novel mutation (c.1243+2T>A) may completely inactivate the original splice-donor site.

## Conclusion and Discussion

In this study, we report the case of a girl clinically diagnosed as EOSRD in her family. This Chinese family were clinically and genetically characterized. The girl had obvious clinical characteristics of EOSRD; however, these characteristics were absent in the fundi of her parents. Clinically, OCT is an important auxiliary diagnosis method to provide in vivo visulization of intraretinal stratification. Disorders of retinal stratification is generally considered to be related with disease. Segmentation of retinal layers is important for diagnosis and analysis of desease [47], [48]. In the grayscale foveal SD-OCT recordings, differences of retinal stratification were very extinct (Figure 3). Based on the method of retinal layers segmentation reported by Ehnes A *et al* [49], the patient’s SD-OCT recordings at the foveal showed altered photoreceptor layers (ELM, IS, ISe, and OS). The boundaries of photoreceptor layers could not be clearly discerned (Figure 3a-2, 3a-3, 3b-1, and 3b-2), and the ELM, IS, ISe and OS layers were not continuous. The ISe, previously named IS/OS junction, could hardly be shown in the OCT. Therefore, we cannot measure the thickness of Ise and RPE; instead, we measured the noncontiguous area of Ise layer (Figure 3g-2), RPE-choroid (Figure 3g-3), and ISe-choroid (Figure 3g-4).

In addition, abnormalities of retinal stratification also included extremely thinned ONL and heavily thinned RPE layer (Figure 3a and 3b). These abnormalities proved the aforementioned relation of disease and retinal stratification disorder. But in her parents’ foveal SD-OCT recordings, the retinal layers could be clearly identified (Figure 3c-2, 3c-3, 3d-1, 3d-2, 3e-2, 3e-3, 3f-1, and 3f-2), and meanwhile the thickness of ISe,RPE, and RPE-choroid could be measured (Figure 3h and 3i). This may be caused by autosomal recessive inheritance (Figure 4). It has been verified that the *RPE65* mutation causes EOSRD [9], [37]. By analysis of mutations in *RPE65* gene in this case, we found that both the mother and the daughter have the mutation (c.1243+2T>A) at the consensus sequence of the splice donor. This mutation uniformly results in splicing errors [50] and is diseasing-causing. However, by ophthalmologic examination, only the daughter has obvious symptoms of EOSRD; we speculate that the daughter’s phenotype may be associated with the combined effects of the c.1243+2T and c.1590C>A mutations.

The entire coding region and adjacent intronic regions of *RPE65* gene were sequenced. Four mutations were identified in the patient, two of which were found novel (c.1243+2T>A and c.1590C>A) and have not been reported before (Table 2). According to previous studies, mutations in *RPE65* gene could cause EOSRD. There are several mutant types in this study:missense, nonsense, splicing site, deletion, insertion, and indel mutation. Eighty-six mutations of *RPE65* gene in patients with LCA have been reported in twenty-four published studies [9]–[32]. Two cases have been reported in 188 Chinese patients [31], [32] containg nine point mutations were identified in *RPE65* gene. These mutations were one insertion mutation (c.1059_1060insG) [51], four missense mutations (c.295G>A, c.997G>C, c.200 T>G, and c.1103A>G) [30], [31], and four polymorphisms (c.643+22C>T, c.1338+20A>C, c.1056G>A, and c.*726_*727insAG) [31]. In this study, four mutations were found in the patient (Table 2), who carried two reported single nucleotide polymorphisms (SNPs) of c.1338+20A>C (rs12534647) and c.1056G>A (rs12145904) [32]. In the SNP database of GenBank, with benign allele was predicted for both c.1338+20A>C and c.1056G>A mutations.

Both c.1243+2T>A and c.1590C>A (F530L) are novel mutations and there are no previous reports. The c.1243+2T>A mutation is a point mutation at consensus sequences at the 5′ end of intron 11 of *RPE65* gene. It has been reported that mutations at consensus sequences uniformly result in aberrant splicing [50]. To confirm the effects of this mutation on splicing, we constructed *RPE65 *minigene from three exons, containing either normal or mutant intron 11 sequences (Figure 5) and investigated their transcripts in 293T cell line by RT-PCR. A normal transcript consisting of three exons (264 bp) was obtained from the wild-type minigene (Figure 6a and b). For the mutant, the transcript is 358 bp in length that is longer than the normal transcript. Sequence analysis showed that the mutant transcript is 94 bp longer than the normal transcript at the 3′ end of exon 11, whose sequence perfectly matches the sequences of intron 11 in *RPE65* gene containing the c.1243+2T>A mutation (Figure 6a and b). These results indicate that the c.1243+2T>A mutation results in aberrant splicing, which is consistent with the result of “splicing site abolised” by Automated Splice Site Analyses. Based on the result of *RPE65 *minigene experiment *in vitro*, the c.1243+2T>A mutation may be pathogenic. Both the mother and the daughter had this mutation; however, the mother showed no symptoms of EOSRD. Since mutations of *RPE65* gene usually cause an autosomal recessive disease, either a single or a compound heterozygous or a homozygous mutation is expected to cause EOSRD. Therefore, the single heterozygous state of the c.1243+2T>A mutation in the mother does not rule out its pathogenicity.

The c.1590C>A change is the second novel mutation in this study. This mutation results in an amino acid transition from phenylalanine to leucine, but it is not detected in patient’s parents. Based on an RPE65 topology diagram, the phenylalanine residue 530 in *RPE65* gene is conserved and located within blade VII of the seven-bladed β-propeller motif in the RPE65 protein [52]. Seven mutations found from LCA and RP patients are reported to be located within blade VII. They are L22P, P25L, G40S, R44Q, H68Y, Y79H, and G528V [14], [15], [17], [24]. In our study, F530L (c.1590C>A) is found to be the eighth mutation in blade VII. Moreover, F530L and G528V are in the same sheet of blade VII. Both biochemical and crystal structure studies on RPE65 show that residues in any sheet of each blade of the propeller structure are essential for RPE65 isomerase activity [5], [52], [53]. In addition, the c.1590C>A (F530L) mutation was also analyzed by the online tools (PolyPhen and SIFT). The potential functional consequence of this nucleotide change was predicted to be “possibly damaging” and “deleterious”. Therefore, the c.1590C>A (F530L) mutation could be responsible for the daughter’s phenotype. It has also been verified to cause the pathogenesis of blade VII in *RPE65* gene, based on its isomerase activity and crystal structure [52], [53]. If there were no other mutant genes involved in ESORD in this family, it could be concluded that this mutation played an important role in the pathogenesis of blade VII in *RPE65* gene.

From the above, we could infer that the c.1590C>A (F530L) mutation together with the c.1243+2T>A mutation is disease-causing mutation. However, *RPE65* is not the only gene associated with EOSRD. Although reports about mutations of the *RPE65* gene are mainly found in EOSRD patients, it is still difficult to say whether the daughter’s phenotype is definitely caused by the two novel mutations, because the mother has one of these novel mutations and shows no symptoms of EOSRD. Either the c.1590C>A (F530L) mutation or other mutated genes in the daughter may be responsible for her phenotype. Thus, further studies should be performed to confirm if there is a definite genotype-phenotype correlation between *RPE65* gene and EOSRD.
